# Ketone Esters Partially and Selectively Rescue Mitochondrial Bioenergetics After Acute Cervical Spinal Cord Injury in Rats: A Time-Course

**DOI:** 10.3390/cells13211746

**Published:** 2024-10-22

**Authors:** Oscar Seira, HyoJoon (David) Park, Jie Liu, Michelle Poovathukaran, Kieran Clarke, Robert Boushel, Wolfram Tetzlaff

**Affiliations:** 1 International Collaboration on Repair Discoveries (ICORD), University of British Columbia, Vancouver, BC V5Z 1M9, Canada; jliu@icord.org (J.L.);; 2 Department of Zoology, University of British Columbia, Vancouver, BC V6T 1Z1, Canada; hyojoon@student.ubc.ca; 3 Department of Physiology, University of Oxford, Oxford OX1 2JD, UK; kieran.clarke@dpag.ox.ac.uk; 4 School of Kinesiology, University of British Columbia, Vancouver, BC V6T 1Z1, Canada; robert.boushel@ubc.ca

**Keywords:** D-β-hydroxybutyrate, metabolism, dietary treatment, respirometry, neurotrauma

## Abstract

Spinal cord injury (SCI) pathology and pathophysiology can be attributed to both primary physical injury and secondary injury cascades. Secondary injury cascades involve dysregulated metabolism and energetic deficits directly linked to compromised mitochondrial bioenergetics. Rescuing mitochondrial function and reducing oxidative stress are associated with neuroprotection. In this regard, ketosis after traumatic brain injury (TBI), or after SCI, improves secondary neuropathology by decreasing oxidative stress, increasing antioxidants, reducing inflammation, and improving mitochondrial bioenergetics. Here, we follow up on our previous study and have used an exogenous ketone monoester, (R)-3-hydroxybutyl (R)-3-hydroxybutyrate (KE), as an alternative to a ketogenic diet, focusing on mitochondrial function between 1 and 14 days after injury. Starting 3 h following a cervical level 5 (C5) hemi-contusion injury, animals were fed either a standard control diet (SD) or a ketone ester diet (KED) combined with KE administered orally (OKE). We found that mitochondrial function was reduced after SCI at all times post-SCI, accompanied by reduced expression of most of the components of the electron transport chain (ETC). The KE rescued some of the bioenergetic parameters 1 day after SCI when D-β-Hydroxybutyrate (BHB) concentrations were ~2 mM. Still, most of the beneficial effects were observed 14 days after injury, with BHB concentrations reaching values of 4–6 mM. To our knowledge, this is the first report to show the beneficial effects of KE in rescuing mitochondrial function after SCI and demonstrates the suitability of KE in ameliorating the metabolic dysregulation that occurs after traumatic SCI without requiring a restrictive dietary regime.

## 1. Introduction

Spinal cord injury (SCI) pathophysiology can be attributed to either primary physical injury or secondary injury cascades. Secondary injury cascades include ischemia, energy failure, oxidative stress, ionic imbalance, excitotoxicity, lipid peroxidation, neuroinflammation, and cell death that lead to further metabolic dysregulation, spinal cord damage, and lack of recovery [1]. It is clear that a better understanding of the injury mechanisms following SCI is essential for the development of therapies to increase neuroprotection, restore metabolic function, and promote functional recovery.

Dysregulation of mitochondrial function has been closely linked to numerous neurological diseases and disorders [2,3]. Metabolism and cellular energetics are severely affected due to mitochondrial dysfunction after SCI ([4], for review), and targeting mitochondrial function might have an important role in promoting recovery after SCI [5,6,7,8]. Progress has been made in studying and understanding the role and use of dietary interventions to treat metabolic dysregulation in neurotrauma events [9,10,11,12,13]. In this regard, ketosis after traumatic brain injury (TBI) or after traumatic spinal cord injury (SCI) improves secondary pathology by decreasing oxidative stress, increasing antioxidants, reducing inflammation, and improving mitochondrial bioenergetics [13,14,15,16,17,18,19].

In this study, we follow up on our previous work showing that ketosis induced by a ketogenic diet (KD) was able to mitigate mitochondrial dysfunction 7 days post-SCI in rats [15]. KD is highly restrictive and may not be suitable for people with SCI. The ketone body, D-β-hydroxybutyrate (BHB), has multiple mechanisms of action ranging from metabolite for mitochondrial energy production to anti-inflammatory receptor ligand, signaling molecule, and epigenetic modifier [13,20,21,22]. Circulating BHB can also be increased using exogenous ketone esters (KEs). In particular, the (R)-3-hydroxybutyl (R)-3-hydroxybutyrate ketone monoester (∆G^®^) can elevate BHB for extended periods in humans at higher levels than those seen with a KD, without side effects and without sodium overload [20,23].

To assess the use of exogenous ketones as an alternative to a KD and to evaluate effects on mitochondrial function in a temporal manner, we focused on the acute–subacute phases of SCI (1 day post-injury; to 14 days post-injury) during which the spinal cord undergoes the most dramatic metabolic changes [24]. Following injury, animals were fed either a standard control diet (SD) or a ketone ester diet (KED) combined with KE administered orally by gavage to achieve robust BHB concentrations of 2–6 mM. KE rescued some bioenergetic parameters of mitochondrial function at early timepoints, but most of the beneficial effects were observed 14 days after treatment.

To our knowledge, this is the first study to investigate the suitability of an exogenous ketone to counteract metabolic dysregulation and mitochondrial dysfunction at multiple timepoints during the acute and subacute phases of traumatic SCI. Our results highlight the beneficial role of KE immediately and for at least up to 14 days after trauma.

## 2. Materials and Methods

### 2.1. Experimental Design, Animal Usage, SCI, and Treatments

All procedures were conducted according to the guidelines of the Canadian Council for Animal Care and overseen by the University of British Columbia Animal Care Committee. We used 51 adult male Sprague–Dawley rats from the Harlan Breeding Laboratory. See the embedded table for details on animal usage.


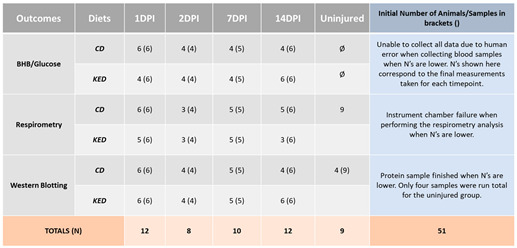


A C5 hemi-contusion injury was performed as we described previously in JoVE [25] (150Kdyn) using the Infinite Horizon impactor (Precision Systems and Instrumentation, LCC, Brimstone, VA, USA). Rats were singly housed in the first 3 days following surgery (including surgery day. Once the rats recovered from surgery, they were group housed. Animals were provided with food pellets and transgel in the cage bottom for the first week following surgery. Animals were given subcutaneous fluids (Ringer’s Lactate twice daily—10 mL) and received Buprenorphine (0.03–0.05 mg/kg, subcutaneously) three times per day for three days (including the day of surgery). During these daily checks, the animals’ bladders were checked, and special care and attention were paid to the look of the animal’s health and signs of pain: (1) Rat Grimace Score (RGS), (2) look of fur (smoothly groomed, or messy/dirty, piloerection), (3) porphyrin (rats only), (4) autophagy (especially on the forepaws in the first few days, (5) surgical incision site, and (6) behavior. On post-injury day 3, the monitoring decreased to twice daily unless there were obvious problems such as bladder issues or signs of pain (RGS/MGS above 0, piloerection, hunching, and/or social isolation). At this time, the animals were still weighed once daily, given ringers (if the animal had lost more than 10% of its pre-surgery weight), and cages still had food pellets, treats, and transgel placed on the bottom of the cage.

In the absence of other health-related outcomes, the animals were weighed and closely inspected once daily for the remainder of the first week post-surgery. The monitoring was only reduced to once/day if the animal had no other health complications. The animals continued to be checked and weighed daily until they returned to within 5% of pre-surgery weight and were free of health concerns. After that point, we limited the weighing and thorough inspections to once a week for the duration of the experiments. During that time, the animals are still looked in on daily (i.e., quick visual inspection of animals in cage) by staff at our holding facility.

### 2.2. KE Administration

The ketone ester (KE) was provided by TdeltaS Global Inc. (Orlando, FL, USA; ∆G^®^ www.deltaGketone.com). The KED and control diet (CD) were prepared by Dyets Inc. (Bethlehem, PA, USA) using the formulation from the Veech lab as previously described [26]. Animals were randomly grouped and exposed to food for 48 h, then put back to normal chow for 4–5 days before surgery day. Animals that were exposed to either of the diets were treated with that same diet after SCI. Starting from 3 h post-injury, the rats were treated with oral gavage of water or ketone esters while given ad libitum access to either a KED (i.e., a control diet with blended in ketone esters) or a control diet (CD). In addition, oral gavage was administered for the first 3 days every 8 h, then every 12 h for 7 days, and later every 24 h for the final 14 days. For the oral ketone ester (OKE), rats were gavaged with either 1 mL of KE or water (controls) mixed 1:1 with Boost^®^ Nutritional Supplement (total volume per gavage = 2 mL). Boost^®^ was used to help mitigate weight loss following injury and improve overall animal health without masking the effects of the KE. (For detailed nutritional information of Boost^®^, visit BOOST Chocolate Meal Replacement|madewithnestle.ca.) This oral gavage treatment would result in an approximate βHB dose of 5 g/kg/oral gavage. The KE content was 0.125 g/1 g food, and as our rats ate around 15–20 g per day, the KE intake was approximately 1.9 to 2.5 g or 1.8 to 2.3 mL (KE density = 1.0731 g/mL at 22 °C).

### 2.3. Blood BHB Levels

**Figure 1:**
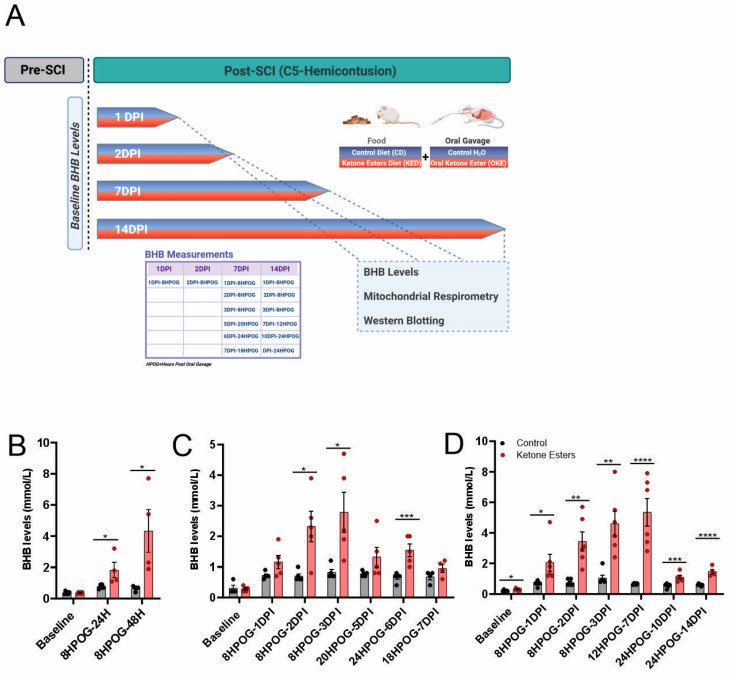
Experimental plan and BHB levels. (**A**) Experimental timeline showing the final outcome measurements of the study: BHB levels, mitochondrial function, and Western blot analysis. Created with BioRender.com. (**B**–**D**) BHB levels measured at 24 h, 48 h, and at several hours post-oral gavage (HPOG) throughout the 1 and 14 days post-SCI cohorts. Baseline BHB values correspond to uninjured (or pre-SCI) animals. (See experimental timeline in (**A**) for details.) Baseline (CD, n = 6, KED, n = 6); 24 h (CD, n = 6, KED n = 4); 48H (CD, n = 6, KED n = 4); 7DPI (CD, n = 5–4, KED n = 5–4); 14DPI (CD, n = 6, KED n = 6–4). Multiple *t*-tests. (* *p* < 0.05, ** *p* < 0.01; *** *p* < 0.001; **** *p* < 0.0001). All data are mean ± SEM.

Ketone levels were measured from blood obtained by tail poking using Precision Xtra Blood Ketone Test Strips (Diabetes Express). Baseline measurements in Figure 1 represent measurements before SCI (uninjured animals). The separation between control and ketone ester groups exists due to the food pre-exposure that took place before the injury.

### 2.4. Mitochondrial Respiration, Protein Extraction, and Western Blots

Rats were euthanized at various times post-injury with an overdose of chloral hydrate injected intraperitoneally. Rats were perfused with cold PBS, and the injury epicenter (approximately half the cord, 5 mm in length) was harvested.

Mitochondrial respiration in permeabilized spinal cord segments was measured using high-resolution respirometry (Oroboros Instruments, Innsbruck, Austria). Spinal cord tissue was permeabilized with saponin at 5 ug/mL for 20′ in the shaker with gentle agitation. Tissue was washed for 10′ with respiration media. Respirometry experiments were performed at 37 °C in respiration medium containing EGTA (0.5 mM), MgCl_2_ (3 mM), K-lactobionate (60 mM), taurine (20 mM), KH_2_PO_4_ (10 mM), HEPES (20 mM), sucrose (110 mM), and BSA (1 g/L), with the addition of mitochondrial substrates and inhibitors to measure coupled and uncoupled respiration, flux through Complexes I and II, and maximal respiration of the electron transport chain (ETC). Mitochondrial respiration was expressed as weight-specific oxygen flux (pmolO_2_·s^−1^·mg^−1^). Chamber oxygen levels were maintained between 240 and 400 nmol·mL^−1^ to avoid O_2_ limitation. As previously described in our lab [15], basal respiration was first measured, then malate (2 mM), pyruvate (5 mM), and glutamate (10 mM) were added to measure LEAK respiration (respiration due to proton leakage and the circuit of electrons and cations that is not dependent on ATP synthase activity). ADP (2.5 mM) was then added to stimulate NADH-dependent coupled respiration through Complex I, followed by oligomycin (2.5 μM) to measure LEAK respiration at high membrane potential during inhibition of ATP synthase. Complete, non-physiological uncoupled respiration of the ETC was measured with the titration of carbonyl cyanide-p-trifluoromethoxyphenylhydrazone (CCCP) (0.5 + 0.5 μM). Succinate (10 mM) was added to measure reduced flavin adenine dinucleotide (FADH2)-dependent Complex II respiration followed by Complex I inhibition with rotenone (0.5 μM). Residual oxygen consumption (ROX) attributed to non-mitochondrial respiration was measured with the addition of Antimycin A (2.5 μM) to inhibit Complex III of the ETC. Finally, ascorbate (2 mM) and N,N,N′,N′-tetramethyl-p-phenylenediamine dihydrochloride (TMPD) (0.5 mM) were added to measure the isolated respiratory rate of cytochrome oxidase (Complex IV respiration).

Protein lysates from the same samples previously used during the respirometry assays were prepared using plastic Dounce homogenizers to disrupt tissue in Tris-EDTA SDS lysis buffer (0.01 M Tris-HCl (pH 8, VWR, Missisauga, ON, Canada)), 1 mM EDTA (Ambion, ThermoFisher Scientific, Burnaby, BC, Canada), 0.1% SDS (VWR, Missisauga, ON, Canada), cOmplete™, Mini, EDTA-free Protease Inhibitor Cocktail (Roche, Mississauga, ON, USA), 100 mM NaF (ThermoFisher Scientific), 25 mM β-glycerophosphate (MilliporeSigma, Toronto, ON, Canada), 10 mM Pyrophosphate (ThermoFisher Scientific, Burnaby, BC, Canada), and 200 µM Orthovanadate (ThermoFisher Scientific, Burnaby, BC, Canada)). Samples were spun at 14,000 rpm, and an aliquot of supernatant was taken for Western blotting. OXPHOS cocktail containing 5 mouse monoclonal antibodies against CI subunit NDUFB8 (ab110242), CII-30kDa (ab14714), CIII-Core protein 2 (ab14745), CIV subunit I (ab14705), and CV alpha subunit (ab14748) and a polyclonal antibody against actin (Proteintech, Rosemont, IL, USA, ref. 20536.1) were used.

### 2.5. Statistical Analysis

Data are presented as mean values, and error bars indicate ± SEM, as noted. All statistical analyses were performed using the Prism 8.0.1 software (http://www.graphpad.com, accessed on 1 March 2018). Information about the tests used can be found in the figure legends. All data were obtained from one cohort of animals.

## 3. Results

### 3.1. A Ketone Ester Diet (KED) in Combination with Orally Administered Ketone Esters (OKEs) Induces Ketosis Acutely After SCI in Rats

We first examined the effects of the ketone ester (KE) on raising BHB levels at 1DPI, 2DPI, 7DPI, and 14DPI (Figure 1A). The injured untreated group consistently revealed some minor but significant increase after injury, which is likely due to post-injury reduction in food intake and stress (Figure 1B–D). BHB levels were significantly higher in the KE-treated groups compared to the injured untreated groups for all timepoints except at the 7DPI timepoint of the 7-day group; here, there was a delay in the time where the BHB measurements were taken (18HPOG vs. 12HPOG), and two animals (one per group) had to be excluded for technical reasons, resulting in a lower N and increased variability (Figure 1C). The equivalent timepoint in the cohort kept alive for two weeks (12HPOG-7DPI) showed the expected significant increase. The smaller differences between injured untreated and treated animals at 10 and 14 days after injury are due to the fact that these had been taken at 24HPOG, reflecting ketone metabolism. Collectively, these data show that ketone levels continued to be sustained throughout the experiment. Baseline values between the groups differed in the 14DPI cohort; however, this difference was small, and the levels were below the known thresholds for ketone effects of 0.5 mmol/L (Figure 1D).

### 3.2. Ketone Esters Selectively Improve Mitochondrial Respiration at 1 Day and 14 Days Post-SCI

**Figure 2:**
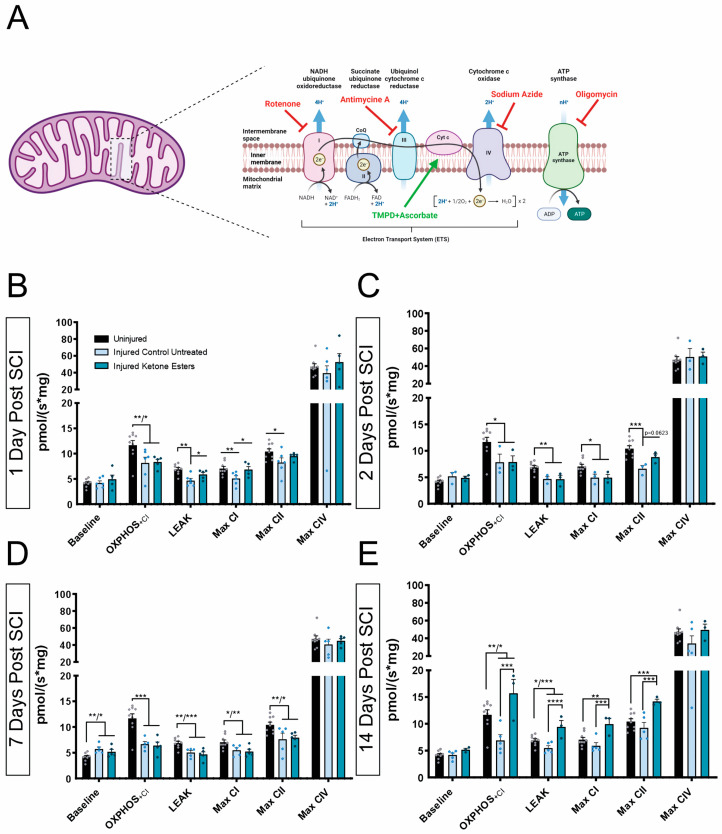
Mitochondrial respiration. (**A**) Schematic representation of the mitochondrial electron transport chain (ETC). Adapted from “Electron Transport Chain” by BioRender.com. (**B**–**E**) Quantification of mitochondrial respiration by high-resolution respirometry showing oxygen flux values of the respiratory states. OXPHOS: representing the maximum oxidative phosphorylation in the presence of metabolic substrates after the addition of ADP; LEAK respiration (proton leak): mitochondrial respiration rate in the presence of an ATP synthase inhibitor (oligomycin); Complex I (CI) maximum respiration: maximum respiration measured after addition of the uncoupler carbonyl cyanide m-chlorophenyl hydrazone (CCCP), causing a depolarization of the cell membrane and leading to an increase in respiration; Complex II (CII) maximum respiration: maximum respiration measured after inhibition of CI with rotenone and the subsequent addition of its substrate, succinate; Complex IV (CIV) maximum respiration: maximum respiration estimated after inhibition of Complex III with Antimycin A, and the subsequent addition of ascorbate and N,N,N′,N′-tetramethyl-p-phenylenediamine (TMPD) as proton donor substrates. Finally, NaN3 (sodium azide) was added to block the respiratory chain by blocking ATPase and leaving the fraction of total respiration that is non-mitochondrial. This value is subtracted from the Complex V (CV) maximum respiration in order to estimate the real mitochondrial CIV respiration. Uninjured (n = 9); 1DPI (CD, n = 6, KED n = 5); 2DPI (CD, n = 3, KED n = 3); 7DPI (CD, n = 5, KED n = 5); 14DPI (CD, n = 5, KED n = 3). One-way ANOVA, Fisher’s LSD post hoc test. (* *p* < 0.05, ** *p* < 0.01; *** *p* < 0.001; **** *p* < 0.0001). All data are mean ± SEM.

Mitochondrial respirometry is a commonly used method to evaluate mitochondrial function and oxidative phosphorylation (OXPHOS) in mitochondria, cells, and tissues [27]. This methodology enables assessment of the overall function of mitochondria in a specific tissue, as well as discrete functional components and characteristics of the electron transport chain (ETC) (Figure 2A) that may be dysfunctional as a result of trauma or pathological disorders.

To evaluate the possible beneficial effects of ketone esters (KEs) in modulating the metabolic distress observed acutely after spinal cord trauma, we performed a respirometry study of nervous tissue located at the epicenter of the injured cervical spinal cords at 1 day, 2 days, 7 days, and 14 days (Figure 2B–E). We followed the same titration protocol we previously used in our ketogenic diet study [15], modified after [28] (see Section 2). Basal mitochondrial respiration (oxygen consumption at baseline) was similar between the different timepoints, except for both injured-treated spinal cords at 7 days, which showed a small but significant elevation when compared to the uninjured group (Figure 2B). As expected from our previous study [15], an overall decline in mitochondrial respiration (i.e., OXPHOS+CI, LEAK, Max CI, Max CII) was observed in the injured untreated animals at 1, 2, and 7 days post-injury (Figure 2B–E). Yet, at 14 days post-injury, maximum respiration via Complex I and Complex II was no longer significantly decreased after injury when compared to the uninjured spinal cords (Figure 2E).

Maximum oxidative phosphorylation in the presence of metabolic substrates after the addition of ADP (OXPHOS + Cl) was significantly higher with KE treatment only at 14 days after injury compared to the untreated injured and uninjured groups. KE treatment reverted the decrease in LEAK respiration, which is measured after oligomycin addition to block ATP synthase activity, at 1 and 14 days (Figure 2B–E).

In addition, KE treatment rescued maximum mitochondrial respiration via Complex I (Max CI) at 1 day and 14 days after injury (Figure 1B,E). Oxygen consumption due to respiration through Complex II, measured in the presence of substrate succinate and the Complex I inhibitor rotenone, was not different between the injured KE-treated group and the untreated injured group after injury at 1, 2, and 7 days post-SCI (Figure 2C,D), but it was increased at 14 days.

Thus, at 14 days, the treatment of the injured group with KE showed a significant increase in maximum oxidative phosphorylation (OXPHOS + Cl), LEAK respiration, and maximum Complex I (CIu) and Complex II (CIIu) uncoupled respiration when compared to the injured untreated group (Figure 2E). Interestingly, the treatment with KE led to an overall increase in mitochondrial respiration that not only rescued the mitochondrial bioenergetic dysfunction associated with the injury but was also able to significantly increase the oxygen consumption to levels that were higher than the ones observed in the uninjured group (Figure 2E).

No differences in the isolated respiration rate of cytochrome c oxidase (Complex IV) were seen among all groups at any timepoint (Figure 2B–E).

### 3.3. Ketone Esters Selectively Modify the Relative Levels of OXPHOS Complexes of the Electron Transport Chain (ETC)

Changes in mitochondrial gene expression have been linked to changes in mitochondrial function after neurotrauma [29]. In fact, we demonstrated that the treatment with a KD led to an increase in relative levels for some of the OXPHOS complexes that are part of the electron transport chain (ETC) in the spinal cord after injury via protein analysis [15].

**Figure 3:**
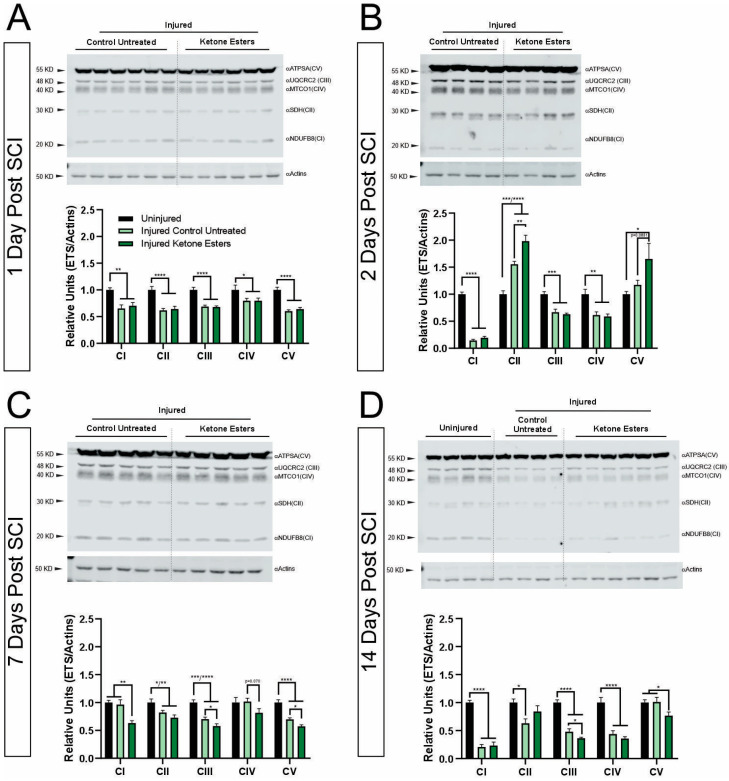
Western blotting analysis of protein levels of the 5 ETS complexes at 1, 2, 7, and 14 days post-SCI. (**A**–**D**) Western blots of total OXPHOS proteins and subsequent quantifications showing an overall decrease in relative levels for most complexes and for all timepoints analyzed, with the exception of CII and CIV at 2 days, CI and CIV at 7 days, and CV at 14 days. Note the increase in the protein levels for CII and CIV at 2 days, as well as decreases in relative levels in CI, CIII, CIV, and CV at 7 days, and CIII and CV at 14 days in the KE versus controls. Uninjured n = 4; 1DPI (CD n = 6, KED n = 6); 48H (CD, n = 4, KED n = 4); 7DPI (CD, n = 5, KED n = 5); 14DPI (CD, n = 4, KED n = 6). One-way ANOVA, Fisher’s LSD post hoc test (per complex). (* *p* < 0.05, ** *p* < 0.01; *** *p* < 0.001; ***** *p* < 0.0001). All data are mean ± SEM.

To further elucidate if the use of KE might have similar effects to those observed with a KD and whether the data from our mitochondrial respirometry could be associated with changes in relative levels of those complexes, we performed individual Western blotting analysis on tissue homogenates from the epicenter of the injury for each of the OXPHOS complexes (Figure 3).

The quantification of the bands revealed that at 1 day post-SCI, there was a general and significant decrease in relative levels of all complexes after injury compared to the uninjured spinal cords. However, the treatment with KE did not reverse the injury effect (Figure 3A). Similar to the 1 day post-SCI, quantification of the 2 days post-SCI blots showed significantly lower relative levels of Complexes I, III, and IV after injury, while Complexes II and V did not appear to change at this timepoint. It was interesting that Complex II relative levels significantly increased after injury with the KE, resulting in an even larger increase in relative levels compared to the injured untreated group. A trend toward an increase in Complex V with KE treatment was also found (Figure 3B). The band quantifications at 7 days post-SCI revealed a significant decrease in relative levels of Complexes II, III, and V (Figure 3C) after injury. Interestingly, the relative levels of Complexes I and IV remained at the same levels as the uninjured cords. Regarding the treatment effects, and surprisingly to us, we found a significant decrease in relative levels after KE treatment of Complexes I, III, and V. Overall, KE caused a decrease in relative levels at 7 days post-SCI (Figure 3C). Lastly, Figure 3D illustrates the quantification for the 14 days post-SCI timepoint. Here, we also found a generalized decrease in relative levels for the OXPHOS Complexes I, II, III, and IV after injury, with the exception of Complex V (Figure 3D). At this timepoint, the KE changes in relative levels were similar to those at 7 days post-SCI. Thus, Complexes III and V relative levels were significantly reduced after treatment compared to the injured untreated group (and also to the uninjured group regarding Complex V). No significant differences were found in the levels of expression between KE and injured untreated for Complexes I, II, and IV. Similar to our mitochondrial respirometry data, a single uninjured group was used as non-injured tissue control and to normalize all quantification data (see uninjured blot in Figure 3D). It should provide a concise and precise description of the experimental results, their interpretation, and the experimental conclusions that can be drawn.

## 4. Discussion

In this study, we demonstrated that ketone ester (KE) can partially mitigate mitochondrial dysfunction after SCI and could be used as an alternative therapeutic intervention to ketogenic diets.

We sought to evaluate the use of KE in restoring energy metabolism following SCI and to investigate whether KE might potentially mimic some of the beneficial effects on mitochondrial function that we found using a ketogenic diet (KD) [15]. Based on the previous literature, we knew that those initial days after injury are critical in regard to cell bioenergetics dysregulation [8,28], so an intensive treatment regime was designed (Figure 1A). With a certain degree of variability, we were able to induce ketosis in the animals at all timepoints studied. BHB levels usually peak between 3 and 7 days post-treatment and decline thereafter. This decline might be associated with cell up-regulation of some receptors, such as the monocarboxylate transporters (MCTs) [30]. MCTs have been identified as transporters of lactate, pyruvate, and ketone bodies [31]. Some of these transporters are expressed not only in the central nervous system (MCT1, MCT2, and MCT4) [32] but in other organs and tissues such as skeletal muscle, liver, and fat tissue [33]. MCTs can be regulated in tissues in response to changes in production or availability of their targeted metabolites [34,35]. For example, diet-induced ketosis has been shown to increase MCT1 levels in the rat brain [30] and in the injured spinal cord [36]. Hence, we hypothesized that there may be an adaptive mechanism that explains the reduction in BHB levels in the bloodstream over time, in which an increase in circulating BHB can increase the expression of MCTs in some tissues, leading to a rapid increase in BHB uptake by the cells.

Our mitochondrial respirometry results showed that KE selectively increases mitochondrial ETC activity in the spinal cord after SCI; increases were only observed at 24H and 14 days post-SCI. In particular, KE led to changes in Complex I and LEAK respiration at 24 h and changes in Complexes I and II, OXPHOS +CI, and LEAK respiration at 14 days post-SCI. Although this approach is widely used and very relevant to assess the overall energetic status of the cord after trauma, determining whether KE rescues mitochondrial function differentially in different cell types (i.e., astrocytes, oligodendrocytes, and neurons) would be of great interest. Recent work by Koppel and collaborators demonstrated that BHB preferentially enhances neuron over astrocyte respiration in a naïve state [37]. Temporally, mitochondrial dysfunction has been shown to start as early as 2 h after SCI and continue until 24 h after SCI [28]. Our measurements obtained beyond the 24 h mark show that, in fact, mitochondrial dysregulation continues for at least 14 days after SCI. This suggests that a feasible therapeutic window not only needs to start early, as previously suggested [28], but may also need to be extended for at least 14 days following injury. Contrary to what we observed using a KD [15], the treatment with KE did not show significant benefits at 7 days after SCI; however, comparable effects were seen at 14 days after SCI. While no statistical differences between the injury parameters were found between groups (see Supplementary Figure S1), inter-individual variability among animals and cohorts can be expected [38]. Differences in the biochemical composition of the treatments might be some of the key contributing factors leading to those differences at that specific timepoint. Indeed, treatment with KE only supplies one ketone body, β-hydroxybutyrate (BHB), whereas when using a KD, the fats from the diet are broken down in the liver to the three ketone bodies: β-hydroxybutyrate (BHB), acetoacetate (AcAC), and acetone. AcAc can be further converted into BHB [39]. Additionally, AcAc has also been shown to act as a signaling metabolite to promote muscle cell growth [40,41,42], increase mitochondrial function in kidney cells in vitro [43], protect against glutamate toxicity in neurons [44], and improve motor coordination and cognition in mice with Angelman syndrome [45]. Whether AcAc might activate signaling pathways in the CNS that could contribute to the early beneficial effects of the KDs after neurotrauma still needs to be further investigated.

Differences in glycemic control between KD and KE treatments might also be relevant to explain the differences in mitochondrial function rescue observed in this study when compared to our previous KD study. For example, KD has been proven to have a therapeutic effect on glucose levels after TBI [46]. Indeed, high glucose levels have been associated with the induction of mitochondrial dysfunction in cardiac models, retina, and neurons [47,48,49,50]. Moreover, in a comparative study, Modica and collaborators described that the supplementation with ketone diester had no effect on glucose when compared to KD [51]. This may suggest that in our study, the inability of KE to decrease glucose might be accountable for the diminished effects on mitochondrial bioenergetics observed at 7 days post-SCI. Nonetheless, the 14 days post-SCI data indicate that KE can still confer relevant bioenergetic benefits but that these may be slightly different and delayed.

In addition to mitochondrial respiration, we also investigated the relative protein expression levels of all the ETC complexes. Mitochondrial respiration is supported by changes in gene expression, protein translation, protein complex formation, transmembrane transport, enzymatic activity, and metabolite levels; how these processes interact with each other and adapt to changes, including nutrient or substrate availability, is poorly known and of great interest in the field [52,53]. Interestingly, several changes in relative levels were found at 2, 7, and 14 days between KE-treated and injured untreated groups. Specifically, KE seemed to significantly reduce the protein expression of some of the subunits of the complexes when compared to the injured untreated group. Only the 2-day timepoint was an exception, in which we saw an increase in relative levels for Complex II after treatment with KE (Figure 3). Interestingly, at this timepoint, Complex II relative levels in the injured untreated group were also increased compared to the uninjured group. The enhanced or maintained relative levels of complexes after injury may reflect a compensatory mechanism to sustain cellular basal oxygen consumption after an increase in cellular energy demand in the injured spinal cord. Furthermore, although not in a time-dependent manner, variability in the relative levels of the ETC complexes (up- or down-regulation) in different regions of the brain has been previously seen in a model of repeated stress in mild traumatic brain injury (mTBI) [54]. Thus, it would seem reasonable to think that variability in relative levels of the ETC complexes in the spinal cord exists at different timepoints as well.

Even though KE led to changes in relative levels, the increase in activity through Complexes I and II was not correlated with an increase or change in protein expression of the targeted subunits (NDUFB8 and SDH, respectively). This lack of correlation between activity and relative levels of complexes has been previously described in cardiac tissue, in a pressure-overload hypertrophy model in rabbits, in human atrial fibrillation [55,56], and in response to exercise training [57,58].

Proposed mechanisms for the lack of synergy between subunit protein expression and respiratory function are changes in mitochondrial protein degradation and transport between proteins transcribed and translated in the cytoplasm versus those in the mitochondria and post-translational modifications of subunits as a result of changes in oxidation. Unfortunately, we did not measure reactive oxygen species (ROS) production, carbonylation, or mutations of mitochondrial DNA in this study [56,59,60,61,62].

Moreover, the formation of enzymatic supercomplexes between some of the mitochondrial complexes might also be contributing to the lack of correlation between individual complex activity and protein content. In fact, it has been described that these supercomplexes might compensate for complex dysfunction by stabilizing individual complexes such as Complex I to changes in the cell environment (i.e., interestingly, the regulation of these supercomplexes has also been linked to neurological disorders [63]). Furthermore, as mentioned above, mitochondrial respiration efficiency can be modified depending on substrate usage. For example, the amount of supercomplexes formed is reduced when certain fuel sources, such as fatty acids, are preferred in the liver during fasting [64]. Whether similar mechanisms occur in the CNS after dietary treatments (i.e., KE) or are involved in traumatic events is still unknown. To date, at the molecular level, it is still unclear how mitochondrial protein import machineries, respiratory complexes, and mitochondrial components assemble to regulate mitochondrial respiration during adaptive responses to various physiological or pathological contexts [52]. What our data seem to indicate is that the beneficial effects of KE (on OXPHOS, LEAK, and Complexes I and II) might be attributed to the effect of BHB (ketones) as a better and more efficient fuel source than glucose. First, it reduces or mitigates ROS production due to its ability to bypass Complex I, which is the primary source of ROS in the cells [65]. Second, their higher redox potential compared to pyruvate (higher hydrogen-to-carbon ratio) leads to higher ATP production [65]. Future studies assessing the enzymatic activities of individual complexes of the ETC would be central to elucidating if the discrepancies we observed between the relative level of the complexes and the oxygen consumption data might be due to enzymatic functionality rather than expression levels.

## 5. Conclusions

In summary, our study provides evidence that KE partially mitigates mitochondrial dysfunction in the acute and subacute phases after SCI. The rescue of mitochondrial function predominantly affects Complexes I and II. Furthermore, our data show that this improvement might not be fully correlated with changes in relative levels of the different ETC complexes. Overall, the work presented here provides support for the beneficial use of KE as an alternative to KD to treat acute metabolic dysfunction that occurs after SCI.

## Data Availability

Raw data are available upon request to the corresponding authors.

## Supplementary Materials

The following supporting information can be downloaded at https://www.mdpi.com/article/10.3390/cells13211746/s1, Figure S1: Surgical parameters.
