# Supplementary material for: Ketone Esters Partially and Selectively Rescue Mitochondrial Bioenergetics After Acute Cervical Spinal Cord Injury in Rats: A Time-Course
Source: Cells. 2024 Oct 22;13(21):1746. doi: 10.3390/cells13211746 (PMC11545339; doi:10.3390/cells13211746)
Supplement: Supplementary file 1 [file cells-13-01746-s001.zip › cells-3071684-supplementary.pdf]

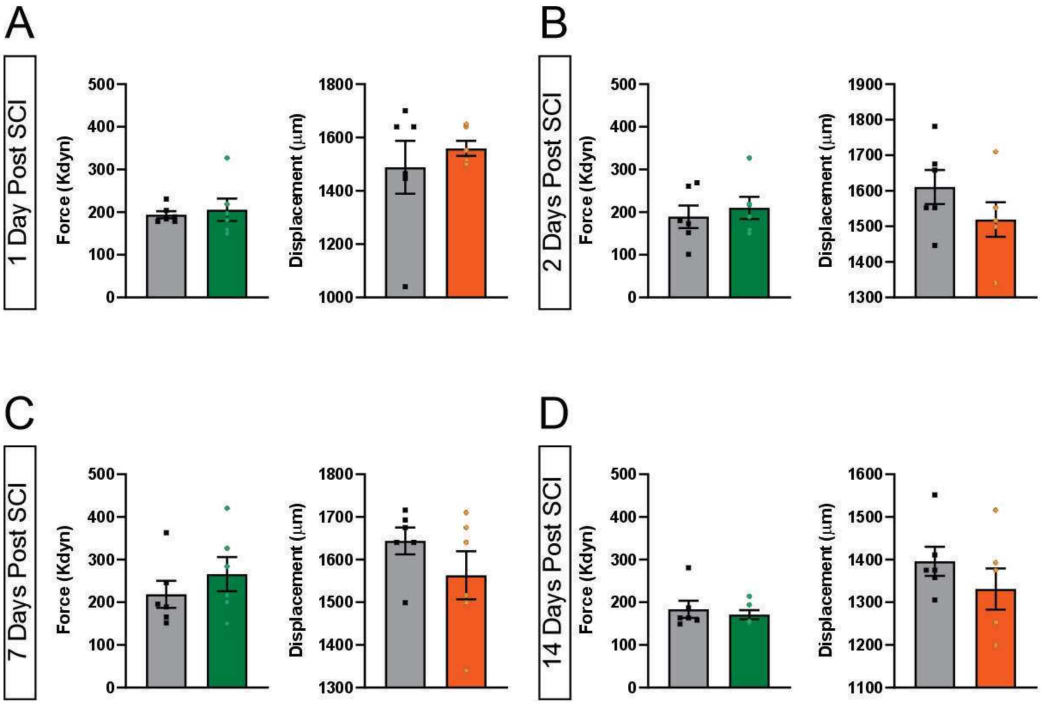

Figure S1. Surgical Parameters. A-D) Force and Displacement parameters were recorded at the time of injury using the IH Impactor software. Grey bars correspond to injured untreated groups, green (force) and orange (displacement) correspond to the KE treated groups. Note: For unknown reasons one of the animals in the 24H injured untreated group shows a much lower displacement despite the cord being confirmed damaged). t-Student test. All data are mean ± SEM.
